# Effects of low-dose clonidine on cardiovascular and autonomic variables in adolescents with chronic fatigue: a randomized controlled trial

**DOI:** 10.1186/s12887-015-0428-2

**Published:** 2015-09-10

**Authors:** Even Fagermoen, Dag Sulheim, Anette Winger, Anders M. Andersen, Johannes Gjerstad, Kristin Godang, Peter C. Rowe, J. Philip Saul, Eva Skovlund, Vegard Bruun Wyller

**Affiliations:** Institute of Clinical Medicine, Medical Faculty, University of Oslo, P.O.Box 1171, Blindern, 0318 Oslo Norway; Department of Anaesthesiology and Critical Care, Oslo University Hospital, P.O.Box 4950, Nydalen, 0424 Oslo Norway; Department of Paediatrics, Oslo University Hospital, P.O.Box 4950, Nydalen, 0424 Oslo Norway; Department of Paediatrics, Lillehammer County Hospital, P.O.Box 104, 2381 Brumunddal, Norway; Institute of Nursing Sciences, Oslo and Akershus University College of Applied Sciences, P.O. Box 4 St., Olavs plass, 0130 Oslo Norway; Department of Pharmacology, Oslo University Hospital, P.O.Box 4950, Nydalen, 0424 Oslo Norway; National Institute of Occupational Health, P.O Box 8149, Dep, 0033 Oslo Norway; Department of Biosciences, University of Oslo, P.O.Box 1066, Blindern, 0316 Oslo Norway; Section of Specialized Endocrinology, Department of Endocrinology, Oslo University Hospital Rikshospitalet, P.O.Box 4950, Nydalen, 0424 Oslo Norway; Department of Paediatrics, Johns Hopkins University School of Medicine, 200 N. Wolfe Street, Baltimore, MD 21287 USA; Department of Paediatrics, Medical University of South Carolina, 169 Ashley Avenue, Charleston, SC 29425 USA; Department of Pharmaceutical Science, University of Oslo, P.O.Box 1068, Blindern, 0316 Oslo Norway; Norwegian Institute of Public Health, P.O.Box 4404, Nydalen, 0403 Oslo Norway; Department of Paediatrics, Akershus University Hospital, P.O.Box 1000, 1478 Lørenskog, Norway

## Abstract

**Background:**

Chronic Fatigue Syndrome (CFS) is a common and disabling condition in adolescence with few treatment options. A central feature of CFS is orthostatic intolerance and abnormal autonomic cardiovascular control characterized by sympathetic predominance. We hypothesized that symptoms as well as the underlying pathophysiology might improve by treatment with the alpha_2A_–adrenoceptor agonist *clonidine*.

**Methods:**

A total of 176 adolescent CFS patients (12–18 years) were assessed for eligibility at a single referral center recruiting nation-wide. Patients were randomized 1:1 by a computer system and started treatment with clonidine capsules (25 μg or 50 μg twice daily, respectively, for body weight below/above 35 kg) or placebo capsules for 9 weeks. Double-blinding was provided. Data were collected from March 2010 until October 2012 as part of The Norwegian Study of Chronic Fatigue Syndrome in Adolescents: Pathophysiology and Intervention Trial (NorCAPITAL). Effect of clonidine intervention was assessed by general linear models in intention-to-treat analyses, including baseline values as covariates in the model.

**Results:**

A total of 120 patients (clonidine group n = 60, placebo group n = 60) were enrolled and started treatment. There were 14 drop-outs (5 in the clonidine group, 9 in the placebo group) during the intervention period. At 8 weeks, the clonidine group had lower plasma norepinephrine (difference = 205 pmol/L, p = 0.05) and urine norepinephrine/creatinine ratio (difference = 3.9 nmol/mmol, p = 0.002). During supine rest, the clonidine group had higher heart rate variability in the low-frequency range (LF-HRV, absolute units) (ratio = 1.4, p = 0.007) as well as higher standard deviation of all RR-intervals (SDNN) (difference = 12.0 ms, p = 0.05); during 20° head-up tilt there were no statistical differences in any cardiovascular variable. Symptoms of orthostatic intolerance did not change during the intervention period.

**Conclusions:**

Low-dose clonidine reduces catecholamine levels in adolescent CFS, but the effects on autonomic cardiovascular control are sparse. Clonidine does not improve symptoms of orthostatic intolerance.

**Trial registration:**

Clinical Trials ID: NCT01040429, date of registration 12/28/2009.

## Background

Chronic Fatigue Syndrome (CFS) is a disabling condition with unknown pathophysiology. In adolescents, prevalence has been estimated from 0.1 to 2.4 % depending on definition of CFS and method of estimation [1, 2]. Apart from a single trial of intravenous immunoglobulin in adolescents with CFS [3], no pharmacotherapy has proven beneficial in this patient population.

Orthostatic intolerance is common with a prevalence of more than 25 % in adults with CFS [4], and more than 90 % in children with CFS [5, 6]. Previously, dysregulation of autonomic cardiovascular control has been demonstrated in adults as well as adolescents, characterized by increased sympathetic and decreased parasympathetic nervous activity [7–10]. This autonomic imbalance might reflect alteration of central control mechanism [11, 12], and provide a target for pharmacotherapy [7, 13].

Clonidine is a centrally acting agonist to the presynaptic alpha_2A_ receptor, thereby attenuating sympathetic nervous activity and enhancing parasympathetic activity, even in low doses [14–16]. Thus, clonidine has well-known antihypertensive properties. A pilot study suggested normalization of cardiovascular variables in adolescent CFS patients receiving low-dose clonidine [17]. However, a single nucleotide polymorphism (SNP) of the alpha_2A_ receptor gene might possible modify the effect of clonidine treatment [18].

The aim of this study was to investigate the effects of low-dose clonidine on autonomic cardiovascular control in adolescent CFS. We hypothesized that clonidine would improve symptoms of orthostatic intolerance and normalize cardiovascular variables and indices of autonomic nervous activity at rest as well as during orthostatic challenges. The study is part of the NorCAPITAL-project (The Norwegian Study of Chronic Fatigue Syndrome in Adolescents: Pathophysiology and Intervention Trial; ClinicalTrials ID: NCT01040429, date of registration 12/28/2009).

## Methods

### Patients

All hospital pediatric departments in Norway (*n* = 20) as well as primary care pediatricians and general practitioners were invited to refer patients aged 12 – 18 years to the national referral center for young CFS patients at Oslo University Hospital. The referring units were equipped with written information for distribution to potential study participants and their parents/next-of-kin. If consent was given, a standard form required the referral unit to confirm the result of clinical investigations considered compulsory to diagnose pediatric CFS according to national Norwegian recommendations (pediatric specialist assessment, comprehensive hematology and biochemistry analyses, chest x-ray, abdominal ultrasound, and brain magnetic resonance imaging). Also, the referring units were required to confirm that the patient a) was unable to follow normal school routines due to fatigue; b) was not permanently bedridden; c) did not have any concurrent medical or psychiatric disorder that might explain the fatigue; d) did not experience any concurrent demanding life event (such as parents’ divorce) that might explain the fatigue; e) did not use prescribed pharmaceuticals (including hormone contraceptives) regularly. A previous demanding life event was not an exclusion criterion. Completed forms were consecutively conveyed to the study center and carefully evaluated by either of two authors (DS or EF). Patients considered eligible to this study were invited to a clinical encounter at our study center after which a final decision on inclusion was made.

In agreement with clinical guidelines [19, 20], this study applied a “broad” case definition of CFS, requiring three months of unexplained, disabling chronic/relapsing fatigue of new onset. We did not require that patients meet any other accompanying symptom criteria. Details of inclusion and exclusion criteria are provided in Table 1.

**Table 1:** Criteria for inclusion and exclusion

	Inclusion criteria	Exclusion criteria
CFS patients	Persisting or constantly relapsing fatigue lasting 3 months or more.	Another current disease process or demanding life event that might explain the fatigue
	Functional disability resulting from fatigue to a degree that prevent normal school attendance	Another chronic disease
	Age ≥ 12 years and < 18 years	Permanent use of drugs (including hormones) possibly interfering with measurements
		Permanently bed-ridden
		Positive pregnancy test
		Pheocromocytoma
		Evidence of reduced cerebral and/or peripheral circulation due to vessel disease
		Polyneuropathy
		Renal insufficiency
		Known hypersensitivity towards clonidine or inert substances (lactose, saccarose) in capsule
		Abnormal ECG (apart from ectopic beats)
		Supine heart rate < 50 beats/min
		Supine systolic blood pressure < 85 mmHg
		Upright systolic blood pressure fall > 30 mmHg
Healthy control subjects	Age ≥ 12 years and < 18 years	Another chronic disease
		Permanent use of drugs (including hormones)

### Study design

All included patients underwent a baseline investigational program at our research unit. Thereafter, they were randomized to 9 weeks of treatment with oral clonidine capsules or placebo capsules in a 1:1 ratio, using a computer-based routine for stratified randomization (block size: 4); 18 months disease duration (the median disease duration in a previous follow-up study [21]) served as the stratification criterion. Because of practical issues, randomization was performed prior to final decision on enrolment; the procedure was carried out by a research nurse not otherwise affiliated with the study. Outcome was assessed by an investigational program identical to the baseline program at week 8 and week 30; in this article, only results from week 8 are reported. Patients and researchers were blinded to treatment allocation at all stages.

Clonidine dosages were 50 μg B.I.D for body weight >35 kg, and 25 μg B.I.D for body weight < 35 kg. Catapresan® 25 μg clonidine hydrochloride tablets (Boehringer Ingelheim, Germany) were enclosed in orange opaque, demolition-restraint lactose capsules (Apoteket Produktion & Laboratorier, Kungens Kurva, Sweden). Identical capsules without Catapresan® were used as placebo comparator. Half the dose was given for the first 3 days of the intervention period in order to minimize adverse introductory effects. Blood samples for clonidine concentration analyses were taken approximately two weeks after start of the intervention, and at the second visit.

NorCAPITAL was approved by the Norwegian National Committee for Ethics in Medical Research and the Norwegian Medicines Agency. Data were collected in the period March 2010 until October 2012. Written informed consent was obtained from all participants, and from parents/next-of-kin if required.

### Investigational program

A one-day in-hospital assessment included clinical examination, blood sampling (antecubital venous puncture), and 20° head-up tilt test (HUT), and always commenced between 7.30 and 9.30 a.m. Patients were instructed to fast overnight and abstain from tobacco products and caffeine for at least 48 h, to bring a morning spot urine sample in a sterile container, and to apply the local anesthetic lidocaine (Emla®) on the skin in the antecubital area one hour in advance. At week 8, CFS patients were told to postpone their prescribed morning study drug dose (clonidine/placebo) until after blood sampling and HUT. All procedures were undertaken in a quiet, warm room in a fixed sequence and by three researchers only (DS, EF and AW). Blood samples were obtained in a fixed sequence from antecubital venous puncture after at least five minutes supine rest in calm surroundings. Samples of oral mucosa were collected for genetic analyses. Following the in-hospital assessment, a self-administered questionnaire was completed.

### Laboratory analyses

The blood samples for plasma norepinephrine (NA) and epinephrine (A) analyses were obtained in vacutainer tubes treated with ethylene glycol tetraacetic acid (EGTA)–Glutathione. The samples were placed on ice for approximately 30 min; thereafter, plasma was separated by centrifugation (3000 rpm, 15 min, 4 °C) and frozen at – 80 °C until assayed. Samples were analyzed for plasma NA and A by high-performance liquid chromatography (HPLC) with a reversed-phase column and glassy carbon electrochemical detector (Antec, Leyden Deacade II SCC, Zoeterwoude, The Netherlands) using a commercial kit (Chromsystems, München, Germany) [22–24]. All samples were measured in singlet, with serial samples from a given individual run at the same time to minimize run-to-run variability. The intra- and interassay coefficient of variation (CV) were 3.9 and 10.8 %, respectively. The detection limit was 5.46 pM.

Urine samples for NA and A analyses were collected in 10 ml universal containers. Immediately after collection the urine was acidified to pH ≈ 2.5, thereafter, stored at 2–8 °C until assayed. Urine treated this way is stable at least 5 days. The analyses were performed consecutively. The same HPLC protocol as for plasma measurement was used for the measurement of urin NA/A. The intra- and interassay coefficient of variation (CV) for urine were 3.9 and 5.2 %, respectively.

The blood samples for clonidine determinations were collected in 4 mL heparin tubes. After centrifugation for 12 min at 1000 g at room temperature, the plasma fraction was frozen at −20 °C until analysis. A slight modification of the method described by Müller et al. [25] was used for plasma clonidine assaying. The assay was validated based on FDA guidelines [26]. The samples were separated on an Alliance HT 2795 HPLC system and detected by a Micromass Quattro micro API MS/MS-instrument. System control, data acquisition and integration were performed by Masslynx software Ver 4.1.2008 (all from Waters, Milford, MA, USA). The MS/MS conditions were optimized by manual tuning during pump-infusion of neat solutions. The assay was set up to quantify from 0.10 μg/L to 5.00 μg/L clonidine in plasma. Quality control samples were included in all sample series, and placed both before and after the patient samples in each analytical run. The median intra assay CV was 1 % at 5 μg/L, 5 % at 0.75 μg/L and 10 % at 0.10 μg/L. The inter assay CV was 6 % at 5 μg/L, 5 % at 0.75 μg/L and 12 % at 0.10 μg/L. Limit of detection, defined as a peak-to-peak signal to noise ratio of 5:1, verified by the Masslynx software, was 0.025 μg/L. Accuracy was 97 % (median) at 5 μg/L, 97 % at 0.75 μg/L, and 107 % at 0.10 μg/L.

The genotyping of the alpha_2A_ receptor single nucleotide polymorphism (SNP) rs1800544 was carried out by predesigned TaqMan SNP genotyping assay (Applied Biosystems, Foster City, CA, USA), using the SDS 2.2 software (Applied Biosystems). As previously described, approximately 10 % of the samples were re-genotyped, and the concordance rate was 100 % [27]. Genotyping was also performed in 68 healthy individuals having the same distribution of gender and age as the CFS patients.

### Head-up tilt-test

Head-up tilt-test (HUT) was performed using an electronically operated tilt table with foot-board support (Model 900–00, CNSystems Medizintechnik, Graz, Austria). Patients were connected to the Task Force Monitor (TFM) (Model 3040i, CNSystems Medizintechnik, Graz, Austria), a combined hardware and software device for noninvasive recording of cardiovascular variables. 5 min was used for supine recordings, after which the participants were head-up tilted to 20° for 15 min. Details of the HUT protocol have been described elsewhere [9]. The feasibility of this protocol for studying adolescent CFS patients has been demonstrated in several previous studies [9, 28]. In particular, the low tilt angle (20°) does not normally precipitate syncope, which is otherwise a common problem among adolescents being subjected to stronger orthostatic challenges [29]. Still, 20° head-up tilt is sufficient to demonstrate hemodynamic alterations and compensatory autonomic responses.

Instantaneous RR intervals (RRI) and heart rate (HR) were obtained from the electrocardiogram (ECG). Continuous arterial blood pressure was obtained noninvasively using photoplethysmography on the right middle finger. Mean arterial blood pressure (BP) was calculated by numerical integration of the recorded instantaneous BP. The recorded value was calibrated against conventional oscillometric measurements of arterial BP on the left arm every five minutes according to the TFM manufacturer’s recommendation. Impedance cardiography with electrodes placed on the neck and upper abdomen was used to obtain a continuous recording of the temporal derivative of the transthoracic impedance (dZ/dt). Beat-to-beat stroke volume was calculated from the impedance signal [30].

Power spectral analysis (frequency-domain method) of HR variability and systolic blood pressure (SBP) variability was automatically provided by the TFM, using an adaptive autoregressive model [31]. Power was calculated in the Low Frequency (LF) range (0.05 to 0.17 Hz), and High Frequency (HF) range (0.17 to 0.4 Hz). In addition, time-domain indices of variability were computed from the RRIs: The standard deviation of all RR-intervals (SDNN), the proportion of successive RRIs with a difference greater than 50 ms (pNN50), and the square root of the mean square differences of successive RRIs (r-MSSD).

Heart rate variability (HRV) is considered an index of autonomic cardiac modulation. In the frequency-domain, vagal (parasympathetic) activity is the main contributor to HF variability, whereas both vagal and sympathetic activity contributes to LF variability [32]. The LF/HF ratio is considered an index of sympathovagal balance. SBP variability is regarded an index of sympathetic modulation of peripheral resistance vessels [33]. For time-domain indices, vagal (parasympathetic) activity is the main contributor to pNN50 and r-MSSD, whereas SDNN is a measure of total variability, analogous to the Total Power index in the frequency domain.

Data from each HUT procedure was exported to Microsoft Excel for further calculations. Beat-to-beat stroke index (SI) was calculated dividing stroke volume by body surface area, and beat-to-beat total peripheral resistance index (TPRI) was calculated as mean BP divided by the product of SI and HR. For each participant, the following epochs of the recordings were chosen: Baseline (270 to 30 s before tilt up) and Early tilt (30 to 270 s after tilt). In each epoch we computed the median value for the conventional cardiovascular variables as well as the indices of HR and SBP variability; this procedure reduces the influence of erroneous outliers, such as ectopic heart beats. Thereafter, the delta values (Early Tilt – Baseline) which are considered indices of the cardiovascular response to orthostatic challenge were computed for each participant. This analytic approach has been proven feasible in several previous report from our group [9–11].

### Questionnaire

The participants received a comprehensive questionnaire consisting of several validated inventories, as has been described in detail elsewhere [28].

The Autonomic Symptom Profile (ASP) [34], which has been used in previous Norwegian CFS studies but which is not validated for the Norwegian language, was slightly modified in order to fit our age group. A composite score reflecting orthostatic symptoms was constructed from 8 single items from the ASP, addressing experiences of dizziness in specific situations (such as rising suddenly from supine position, taking a shower, etc.). The total sum score is from 0 to 8; higher values reflect more pronounced orthostatic problems. In addition, other symptoms related to autonomic cardiovascular control, such as palpitations and pale and cold hands, were charted on a 1–5 Likert scale.

The questionnaire also included the CFS symptom inventory for adolescents [28, 35]. This inventory was used to subgroup the CFS patients according to the 1994 CFS case definition [36].

### Statistics

Determination of sample size is described elsewhere [28]. Outcome of clonidine intervention was assessed by general linear models (ANCOVA) in intention-to-treat analyses, including baseline values as covariates in the model [37]. The net intervention effect was calculated from the parameters of the fitted general linear model. Differential effects in subgroups adhering to the 1994 CFS case definition, genotype of the alpha_2A_ receptor single nucleotide polymorphism (SNP) rs1800544, and sex, were explored by including these variables as interaction terms. Dose–response relationships for patients allocated to clonidine were explored by linear regression analyses. Missing values were imputed as last observation carried forward from the pre-medication test. In order to obtain near-normally distributed variables, ln-transformation was carried out for supine values of LF-HRV, HF-HRV, Total Power-HRV, LF/HF ratio and LF-SBP. Square root transformation was carried out for 20° head-up tilt values of LF-HRV, HF-HRV and Total Power-HRV. Genotype frequency among patients and healthy controls were explored with chi-square analyses.

SPSS statistical software (SPSS Inc., Chicago, IL, USA) was applied for all statistical analyses, and all tests were carried out two-sided. A p-value ≤ 0.05 was considered statistically significant. Corrections for multiple comparisons were not applied.

## Results

**Fig. 1:**
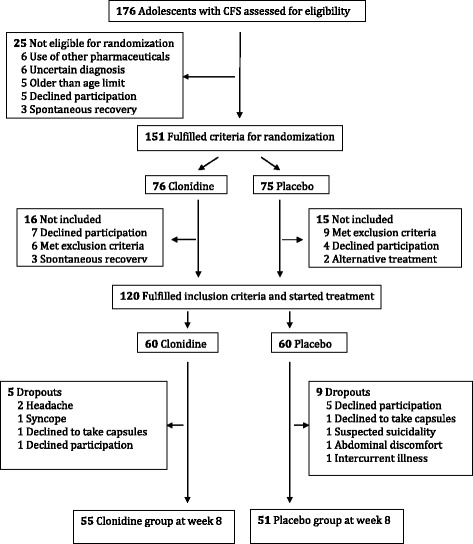
Study flowchart. Study flowchart. A total of 176 adolescents with CFS were assessed for eligibility. Of these, 151 fulfilled randomization criteria, whereas 120 started treatment. At week 8, 106 participants were still participating in the intervention program, 55 in the clonidine group and 51 in the placebo group

A total of 176 CFS patients were referred to the study, of which 151 were eligible for randomization (Fig. 1). A total of 120 patients were enrolled and started treatment; 60 patients in the clonidine group and 60 patients in the placebo group. At week 8, there were 5 dropouts in the clonidine group and 9 dropouts in the placebo group (Fig. 1). Further baseline demographic and clinical characteristics are given in Table 2.

**Table 2:** Background characteristics

	Clonidine (*n* = 60)	Placebo (*n* = 60)
Gender - no. (%)		
Male	13 (22)	21 (35)
Female	47 (78)	39 (65)
Age - years, mean ± SD	15.3 ± 1.5	15.5 ± 1.6
BMI - kg/m^2^, mean ± SD	21.6 ± 4.4	21.5 ± 4.0
Adheres to 1994 CFS case definition - no. (%)		
No	14 (24)	15 (26)
Yes	45 (76)	43 (74)
Genotype ^a^ – no. (%)		
C/C	32 (53)	35 (58)
C/G	25 (42)	19 (32)
G/G	3 (5)	6 (10)
Disease duration - months, median (range)	18 (4 to 72)	18 (5 to 104)
Disease duration – months, mean ± SD	19.4 ± 13.0	23.5 ± 17.0
School absenteism - %, mean ± SD	66 ± 29	64 ± 31
Smokers – more than once a week – no.	1	0

^a^ The alpha_2A_ receptor single nucleotide polymorphism (SNP) rs1800544. C = Cytosine, G = Guanine

At week 8, the clonidine group had statistically significantly lower plasma norepinephrine (*p* = 0.05) and urine norepinephrine/creatinine ratio (*p* = 0.002) as compared to the placebo group (Table 3). At supine rest, the clonidine group had higher heart rate variability in the low-frequency band (LF-HRV, absolute unites) (*p* = 0.007) and as well as higher SDNN (*p* = 0.05) (Table 4). No other significant differences were observed. In particular, symptoms of orthostatic intolerance did not change during the intervention period.

**Table 3:** Outcome of clonidine intervention – symptom scores and catecholamines

	Baseline	Week 8 (during treatment)
*Symptoms scores*		
Orthostatic symptoms – total score		
Clonidine group, mean	3.8	3.5
Placebo group, mean	3.5	3.5
Difference (95 % CI)		−0.05 (−0.5 to 0.4)
p-value (clonidine vs. placebo)		0.84
Palpitations - score		
Clonidine group, mean	2.4	2.2
Placebo group, mean	2.2	2.2
Difference (95 % CI)		0.06 (−0.3 to 0.4)
p-value (clonidine vs. placebo)		0.73
Pale and cold hands - score		
Clonidine group, mean	3.0	2.7
Placebo group, mean	3.0	2.8
Difference (95 % CI)		−0.1 (−0.5 to 0.3)
p-value (clonidine vs. placebo)		0.62
*Catecholamines*		
Plasma norepinephrine - pmol/L		
Clonidine group, mean	2040	1557
Placebo group, mean	1942	1761
Difference (95 % CI)		−205 (−406 to −4)
p-value (clonidine vs. placebo)		0.05
Plasma epinephrine - pmol/L		
Clonidine group, mean	327	291
Placebo group, mean	415	299
Difference (95 % CI)		−8 (−44 to 29)
p-value (clonidine vs. placebo)		0.68
Urine norepinephrine/creatinine ratio - nmol/mmol		
Clonidine group, mean	13.3	9.6
Placebo group, mean	13.7	13.6
Difference (95 % CI)		−3.9 (−6.4 to −1.5)
p-value (clonidine vs. placebo)		0.002
Urine epinephrine/creatinine ratio - nmol/mmol		
Clonidine group, mean	1.7	1.2
Placebo group, mean	1.6	1.6
Difference (95 % CI)		−0.4 (−0.8 to 0.1)
p-value (clonidine vs. placebo)		0.11

Missing values were imputed based on the principle of last observation carried forwards. Thus, all calculations are based on 120 individuals (60 in each intervention group except one to two in each group with missing values at baseline). Means and differences at week 8 are estimated from the parameters of the general linear model

**Table 4:** Outcome of clonidine intervention – cardiovascular variables

	Baseline	Week 8 (during treatment)
*Supine*		
Heart rate - beats/min		
Clonidine group, mean	70	67
Placebo group, mean	72	69
Difference (95 % CI)		−2.0 (−4.1 to 0.1)
p-value (clonidine vs. placebo)		0.06
SBP – mmHg		
Clonidine group, mean	103	104
Placebo group, mean	107	103
Difference (95 % CI)		1.4 (−1.0 to 3.9)
p-value (clonidine vs. placebo)		0.25
MBP – mmHg		
Clonidine group, mean	77	78
Placebo group, mean	80	77
Difference (95 % CI)		1.3 (−0.7 to 3.4)
p-value (clonidine vs. placebo)		0.19
DBP – mmHg		
Clonidine group, mean	65	64
Placebo group, mean	66	63
Difference (95 % CI)		0.8 (−1.0 to 2.7)
p-value (clonidine vs. placebo)		0.37
SI - ml/m^2^		
Clonidine group, mean	47	46
Placebo group, mean	46	46
Difference (95 % CI)		0.2 (−2.1 to 2.4)
p-value (clonidine vs. placebo)		0.86
TPRI - mmHg/L/min/m^2^		
Clonidine group, mean	9.1	9.4
Placebo group, mean	8.9	8.9
Difference (95 % CI)		0.5 (−0.1 to 1.1)
p-value (clonidine vs. placebo)		0.11
SDNN – ms		
Clonidine group, mean	74	78
Placebo group, mean	66	66
Difference (95 % CI)		12.0 (−0.2 to 23.7)
p-value (clonidine vs. placebo)		0.05
r-MSSD – ms		
Clonidine group, mean	79	83
Placebo group, mean	65	70
Difference (95 % CI)		13.1 (−3.2 to 29.5)
p-value (clonidine vs. placebo)		0.11
pNN50 - %		
Clonidine group, mean	40	40
Placebo group, mean	31	38
Difference (95 % CI)		2.2 (−3.0 to 7.3)
p-value (clonidine vs. placebo)		0.40
LF-HRV – nu		
Clonidine group, mean	40	42
Placebo group, mean	43	38
Difference (95 % CI)		3.7 (−0.5 to 8.0)
p-value (clonidine vs. placebo)		0.08
HF-HRV – nu		
Clonidine group, mean	60	58
Placebo group, mean	57	62
Difference (95 % CI)		−3.7 (−8.0 to 0.5)
p-value (clonidine vs. placebo)		0.08
LF-HRV* - ms^2^		
Clonidine group, mean	628	679
Placebo group, mean	451	487
Ratio (95 % CI)		1.4 (1.1 to 1.8)
p-value (clonidine vs. placebo)		0.007
HF-HRV* - ms^2^		
Clonidine group, mean	962	961
Placebo group, mean	600	825
Ratio (95 % CI)		1.2 (0.9 to 1.5)
p-value (clonidine vs. placebo)		0.28
Total Power-HRV* - ms^2^		
Clonidine group, mean	1991	2053
Placebo group, mean	1352	1638
Ratio (95 % CI)		1.3 (1.0 to 1.6)
p-value (clonidine vs. placebo)		0.06
LF/HF-ratio*		
Clonidine group, mean	0.65	0.70
Placebo group, mean	0.75	0.59
Ratio (95 % CI)		1.2 (1.0 to 1.4)
p-value (clonidine vs. placebo)		0.09
LF-SBP – nu		
Clonidine group, mean	39.3	38.0
Placebo group, mean	38.1	36.9
Difference (95 % CI)		1.1 (−3.0 to 5.2)
p-value (clonidine vs. placebo)		0.60
LF-SBP* - mmHgs^2^		
Clonidine group, mean	3.8	3.7
Placebo group, mean	3.0	3.2
Ratio (95 % CI)		1.1 (0.9 to 1.5)
p-value (clonidine vs. placebo)		0.34
*Response to 20° head-up tilt*		
Heart rate - beats/min		
Clonidine group, mean	5.2	4.9
Placebo group, mean	4.8	4.9
Difference (95 % CI)		0.0 (−1.1 to 1.2)
p-value (clonidine vs. placebo)		0.97
SBP – mmHg		
Clonidine group, mean	0.74	−0.59
Placebo group, mean	0.15	−0.01
Difference (95 % CI)		−0.58 (−2.2 to 1.0)
p-value (clonidine vs. placebo)		0.48
MBP - mmHg		
Clonidine group, mean	1.19	0.61
Placebo group, mean	0.94	1.23
Difference (95 % CI)		−0.63 (−2.1 to 0.8)
p-value (clonidine vs. placebo)		0.39
DBP - mmHg		
Clonidine group, mean	1.13	1.2
Placebo group, mean	1.58	1.8
Difference (95 % CI)		−0.59 (−2.0 to 0.8)
p-value (clonidine vs. placebo)		0.40
SI - ml/m^2^		
Clonidine group, mean	−5.9	−4.5
Placebo group, mean	−5.1	−5.3
Difference (95 % CI)		0.9 (−0.4 to 2.1)
p-value (clonidine vs. placebo)		0.17
TPRI - mmHg/L/min/m^2^		
Clonidine group, mean	0.66	0.44
Placebo group, mean	0.60	0.62
Difference (95 % CI)		−0.18 (−0.47 to 0.11)
p-value (clonidine vs. placebo)		0.22
SDNN - ms		
Clonidine group, mean	−5.1	−7.9
Placebo group, mean	−4.4	−0.7
Difference (95 % CI)		−7.2 (−16.0 to 1.6)
p-value (clonidine vs. placebo)		0.11
r-MSSD - ms		
Clonidine group, mean	−18	−24
Placebo group, mean	−16	−17
Difference (95 % CI)		−7.6 (−19.6 to 4.4)
p-value (clonidine vs. placebo)		0.11
pNN50 - %		
Clonidine group, mean	−14	−11
Placebo group, mean	−9	−13
Difference (95 % CI)		1.2 (−3.1 to 5.4)
p-value (clonidine vs. placebo)		0.59
LF-HRV - nu		
Clonidine group, mean	8.3	6.1
Placebo group, mean	6.7	9.2
Difference (95 % CI)		−3.1 (−7.4 to 1.1)
p-value (clonidine vs. placebo)		0.15
HF-HRV - nu		
Clonidine group, mean	−8.3	−6.1
Placebo group, mean	−6.7	−9.2
Difference (95 % CI)		3.1 (−1.1 to 7.4)
p-value (clonidine vs. placebo)		0.15
LF-HRV^#^ - ms^2^		
Clonidine group, mean	−320	−161
Placebo group, mean	−176	−171
n.a.		n.a.
p-value (clonidine vs. placebo)		0.87
HF-HRV^#^ - ms^2^		
Clonidine group, mean	−828	−640
Placebo group, mean	−523	−629
n.a.		n.a.
p-value (clonidine vs. placebo)		0.99
Total Power-HRV^#^ - ms^2^		
Clonidine group, mean	−1107	−790
Placebo group, mean	−668	−736
n.a.		n.a.
p-value (clonidine vs. placebo)		0.78
LF/HF-ratio		
Clonidine group, mean	0.35	0.34
Placebo group, mean	0.44	0.55
Difference (95 % CI)		−0.21 (−0.46 to 0.04)
p-value (clonidine vs. placebo)		0.09
LF-SBP - nu		
Clonidine group, mean	2.5	4.4
Placebo group, mean	3.2	3.7
Difference (95 % CI)		0.7 (−2.4 to 3.8)
p-value (clonidine vs. placebo)		0.66
LF-SBP - mmHgs^2^		
Clonidine group, mean	−2.6	−1.0
Placebo group, mean	−0.6	−0.2
Difference (95 % CI)		−0.7 (−1.7 to 0.3)
p-value (clonidine vs. placebo)		0.17

Missing values were imputed based on the principle of last observation carried forwards. Thus, all calculations are based on 120 individuals (60 in each intervention group). Means and differences at week 8 are estimated from the parameters of the general linear model

For variables annotated with a *, modeling was performed on ln-transformed variables; all means are based on back-transformation of the variables, and ratios instead of differences are reported. For variables annotated with a #, modeling was performed on square root-transformed variables; all means are based on back-transformation of the variables, but neither differences nor ratios can be computed, as indicated with the label n.a. (not applicable). CI = Confidence Interval; SBP = Systolic Blood Pressure; MBP = Mean arterial Blood Pressure; DBP = Diastolic Blood Pressure; SI = Stroke Index; TPRI = Total Periferal Resistance Index; RRI = R-R Interval; HRV = heart rate variability; HF = High Frequency; LF = Low Frequency; SDNN = standard deviation of all RR-intervals; pNN50 = the proportion of successive RRIs with adifference greater than 50 ms; r-MSSD = the square root of the mean square differences of successive RRIs; nu = normalized units; n.a. = not applicable because of square root transformation of variables; n = number of patients, for most variables equal to 60 because of imputation

Urine norepinephrine/creatinine ratio was negatively related to plasma clonidine concentration (*B* = −14.5, *p* = 0.004). TPRI supine (*B* = 4.1, *p* = 0.01), heart rate variability in the low-frequency band supine (LF-HRV, absolute unites) (*B* = 1423, *p* = 0.02) and HRV-Total Power supine (*B* = 4353, *p* = 0.04) were positively related to plasma clonidine concentration. No other dose response-relationships were found.

Subgrouping according to the 1994 CFS case definition, genotype frequency of the alpha_2A_ receptor SNP rs1800544 and sex did not reveal any differential response to the intervention. Also, the genotype frequency was equal among CFS patients and healthy controls (*p* = 0.75).

## Discussion

This study shows that clonidine reduces catecholamine levels in adolescent CFS. However, the effects on cardiovascular autonomic control are sparse, and clonidine does not improve symptoms of orthostatic intolerance.

Previous studies have documented that adult as well as adolescent CFS patients are characterized by enhanced sympathetic and attenuated parasympathetic nervous activity [7, 9, 38, 39]. In particular, CFS patients have increased levels of catecholamines [40, 41] and a sympathetic predominance of cardiovascular autonomic control possibly due to central alterations [9, 11, 42]. In this study, clonidine lowered catecholamine levels as expected. Of note, urine norepinephrine, which is considered an index of sympathetic nervous activity over time [43], decreased dose-dependently.

Clonidine had limited impact on standard cardiovascular variables, both at rest and during orthostatic challenge. This finding was surprising. In previous studies of healthy individuals as well as hypertensive patients, clonidine dosages similar to those applied in this study have been shown to decrease both blood pressures and heart rate, and these alterations of hemodynamics were paralleled by a decrement of catecholamines [15, 44–47]. Furthermore, in healthy subjects, clonidine also attenuates indices of cardiovascular sympathetic nervous modulation (such as LF-HRV), both in supine and sitting positions [44]. In this study, there was a clonidine-mediated increase in LF-HRV at supine rest, as well as a positive relationship between LF-HRV and clonidine plasma concentration. The interpretation of LF-HRV-indices is not straight forward; these results, however, might suggest an enhancement of sympathetic heart rate modulation, resembling the effects of clonidine in essential hypertension [48]. This is in contrast to effects of clonidine in healthy subjects [44]. A previous study suggests early sympathetic baroreceptor activation and diminished baroreceptor reserve in CFS [11]. We speculate that clonidine, by way of reducing sympathetic tone (as evident from the catecholamine-lowering effect), might in fact increase the sympathetic nervous system modulatory effects [49].

Taken together, the findings presented in this study suggest an alteration of clonidine pharmacodynamics in CFS. One possible explanation is genetically determined differences of the alpha_2A_ receptor protein, which is the ligand for clonidine. A single nucleotide polymorphism (SNP) (rs1800544) in the alpha_2A_ receptor gene implies substitution of guanine (G) for cytosine (C) at position 1291, and has functional consequences [18]. However, the genotype frequencies among CFS patients and a comparable group of healthy controls were almost identical, and subgroup analysis based on genotype revealed no differences in response to treatment. Another possible explanation is altered expression of adrenoceptors, as has previously been demonstrated in CFS [50] as well as in other conditions with high levels of catecholamines [51].

The possibility of increased long-term cardiovascular risk in CFS patients remains a concern [52]. In addition to increased sympathetic nervous activity, CFS patients are also characterized by slight inflammatory activation [28] and elevated nocturnal blood pressure and heart rate [53], which in turn are associated with development of atherosclerosis. Further research is warranted to clarify the eventual need of prophylactic measures.

A possible limitation of this study is the wide inclusion criteria and no *a priori*-definition of the degree of school absenteeism necessary to fulfil the diagnostic criteria, which might have obscured results applying to a subgroup only. However, the study population corresponds closely to the population who is diagnosed as CFS by pediatricians; thus, we assume the external validity to be strong. Furthermore, subgrouping based upon the 1994 CFS case definition did not change the results. We have not done subgrouping based on caffeine use. Another limitation of this study is the 4 min epochs used for time-domain analyses of heart rate variability, as opposed to the 5 min epochs recommended [32]. It is considered inappropriate to compare time-domain indices (especially SDNN) obtained from recordings of different durations; while the present study does not violate this principle, caution should be shown when comparing our results to other studies. Strengths of this study include high compliance and low drop-out-rates, and the successful blinding of all (staff and patients) clinically involved in the study.

## Conclusions

Low-dose clonidine reduces catecholamine levels in adolescent CFS. However, the effects on cardiovascular autonomic control are sparse, and clonidine does not improve symptoms of orthostatic intolerance.

## Abbreviations

BP: Blood pressure
CFS: Chronic fatigue syndrome
HF: High frequency
HR: Heart rate
HRV: Heart rate variability
HUT: Head-up tilt test
LF: Low frequency
RRI: Instantaneous RR intervals
SBP: Systolic blood pressure
SNP: Single nucleotide polymorphism
